# Disruption in the balance between apolipoprotein A‐I and mast cell chymase in chronic hypersensitivity pneumonitis

**DOI:** 10.1002/iid3.355

**Published:** 2020-10-04

**Authors:** Yukihisa Inoue, Tsukasa Okamoto, Takayuki Honda, Yoshihisa Nukui, Takumi Akashi, Tamiko Takemura, Minoru Tozuka, Yasunari Miyazaki

**Affiliations:** ^1^ Department of Respiratory Medicine Tokyo Medical and Dental University Tokyo Japan; ^2^ Department of Pathology Tokyo Medical and Dental University Tokyo Japan; ^3^ Department of Pathology Japan Red Cross Centre Tokyo Japan; ^4^ Department of Analytical Laboratory Chemistry, Graduate School of Medical and Dental Sciences Tokyo Medical and Dental University Tokyo Japan

**Keywords:** apolipoprotein A‐I, chymase, hypersensitivity pneumonitis, mast cell

## Abstract

**Background:**

Apolipoprotein A‐I (apoA‐I) has an antifibrotic effect in idiopathic pulmonary fibrosis. Although pulmonary fibrosis is associated with poor prognosis of patients with hypersensitivity pneumonitis (HP), little is known regarding the role of apoA‐I in the pathogenesis of HP.

**Methods:**

Two‐dimensional electrophoresis, immunoblotting, and enzyme‐linked immunosorbent assays were performed for the identification and quantification of apoA‐I in bronchoalveolar lavage fluid (BALF) from patients with acute and chronic HP. To investigate the degradation of apoA‐I, apoA‐I was incubated with BALF. Moreover, the role of apoA‐I in TGF‐β1‐induced epithelial–mesenchymal transition of A549 cells was examined.

**Results:**

The concentration of apoA‐I in the BALF was significantly lower in chronic HP (*n* = 56) compared with acute HP (*n* = 31). The expression level of apoA‐I was also low in the lung tissues of chronic HP. ApoA‐I was degraded by BALF from HP patients. The number of chymase‐positive mast cells in the alveolar parenchyma was inversely correlated with apoA‐I levels in the BALF of chronic HP patients. In vitro experiment using A549 cells, untreated apoA‐I inhibited TGF‐β1‐induced epithelial–mesenchymal transition, although this trend was not observed in the chymase‐treated apoA‐I.

**Conclusions:**

A decrease of apoA‐I was associated with the pathogenesis of chronic HP in terms of pulmonary fibrosis and mast cell chymase attenuated the protective effect of apoA‐I against pulmonary fibrosis. Furthermore, apoA‐I could be a crucial molecule associated with lung fibrogenesis of HP.

## Graphical abstract

A decrease of apolipoprotein A‐I (apoA‐I) was associated with the pathogenesis of chronic hypersensitivity pneumonitis (HP) in terms of pulmonary fibrosis and mast cell chymase attenuated the protective effect of apoA‐I against pulmonary fibrosis. Furthermore, apoA‐I could be a crucial molecule associated with lung fibrogenesis of HP.

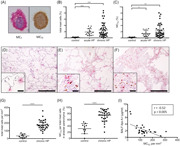

**Abbreviations**
AEC: alveolar epithelial cell
apoA‐I: apolipoprotein A‐I
BALF: bronchoalveolar lavage fluid
ELISA: enzyme‐linked immunosorbent assay
EMT: epithelial–mesenchymal transition
HDL: high‐density lipoprotein
HP: hypersensitivity pneumonitis
IPF: idiopathic pulmonary fibrosis
MC_T_: tryptase‐positive but chymase‐negative mast cells
MC_TC_: tryptase‐positive and chymase‐positive mast cells

## 1 INTRODUCTION

Hypersensitivity pneumonitis (HP) is an immune‐mediated lung disease induced by inhalation of various antigens^1^, ^2^ and is classified into acute and chronic forms.^3^ Patients with acute HP achieve a relatively good prognosis by antigen avoidance, but prolonged exposure to small amounts of the offending antigens induces progression of pulmonary fibrosis with poor prognosis in patients with chronic HP, similar to idiopathic pulmonary fibrosis (IPF).^4^, ^5^, ^6^ Proteomic analysis of bronchoalveolar lavage fluid (BALF) directly reflects pulmonary disorders and is useful for the discovery of biomarkers.^7^ We previously revealed that surfactant protein A, immunoglobulin heavy chain α, α‐2 heat shock glycoprotein, haptoglobin β, and immunoglobulin J chain were increased in BALF from chronic HP patients with usual interstitial pneumonia (UIP) pattern compared with those with fibrotic nonspecific interstitial pneumonia (f‐NSIP) pattern.^8^ However, no studies regarding the differences in the protein profile of BALF between acute and chronic HP have been reported. The distinctive protein profile of BALF in between acute and chronic HP may be considered to reflect the pathogenesis of pulmonary fibrosis in HP.^9^

Apolipoprotein A‐I (apoA‐I), the major component of plasma high‐density lipoproteins (HDLs), plays an important role in regulating normal lipid homeostasis by clearing cholesterol and phospholipids from cells.^10^ ApoA‐I can also regulate inflammatory responses and attenuate the risk of atherosclerotic cardiovascular disease.^11^ Additionally, apoA‐I is expressed in alveolar epithelial cells (AECs) and has anti‐inflammatory effects in various lung diseases.^12^ We previously revealed that the concentrations of BALF apoA‐I were higher in Stage I sarcoidosis compared with Stage IV sarcoidosis, suggesting that BALF apoA‐I might protect against lung inflammation.^13^ ApoA‐I also has antifibrotic effects on pulmonary fibrosis, as previously reported that apoA‐I was decreased in BALF from patients with IPF.^14^ Intranasal administration of apoA‐I also reduced pulmonary fibrosis in a murine model of bleomycin‐induced fibrosis.^14^

Taking these results into consideration, the expression of apoA‐I could be associated with the pathogenesis of chronic HP with fibrosis. However, no studies assess the association between pulmonary fibrosis of chronic HP and the expression of apoA‐I in BALF. Additionally, the dynamics of apoA‐I in the human lung, including its expression and degradation, have not been thoroughly elucidated to date. The aim of this study was to evaluate the dynamics of apoA‐I in patients with acute and chronic HP and to investigate the role of the degradation of apoA‐I using in vitro experiments.

## 2 MATERIALS AND METHODS

### 2.1 Patient selection

All of the recruited patients were diagnosed at Tokyo Medical and Dental University Hospital between January 1993 and December 2013 and informed consent was obtained. The patients fulfilled the diagnostic criteria of acute HP or chronic HP, as described previously.^15^, ^16^ We collected serum and BALF samples from all patients at their first admissions and reviewed their medical records. This study conformed to the Declaration of Helsinki and was approved by the institutional review board at Tokyo Medical and Dental University (approval number: M2017‐031).

### 2.2 Bronchoalveolar lavage (BAL)

We performed BAL using three 50‐ml aliquots of sterile 0.9% saline, as described previously.^17^ The cellular composition of BALF was determined by counting 200 cells in a cytospin smear with Wright's staining and May–Grünwald–Giemsa staining. Lymphocyte phenotypes were analyzed by flow cytometry using monoclonal antibodies against CD4 and CD8.

### 2.3 Pathological assessment

Histological patterns of surgical lung biopsy specimens were evaluated by two pulmonary pathology specialists (T. T. and T. A.) without any prior knowledge of the patient's clinical information. Histological patterns were based on the international classification of idiopathic interstitial pneumonias proposed by the joint American Thoracic Society/European Respiratory Society (ATS/ERS) statement.^18^ When the opinions differed between the two pathologists, the final decision was reached by consensus.

### 2.4 Antibodies

Anti‐human apoA‐I rabbit polyclonal antibody was obtained from ProteinTech. Anti‐human mast cell tryptase mouse monoclonal antibody was obtained from Chemicon International Inc. Anti‐human mast cell chymase (CC1) mouse monoclonal antibody and anti‐human β‐actin antibody were obtained from Abcam. Anti‐E‐cadherin rabbit monoclonal antibody, anti‐N‐cadherin rabbit monoclonal antibody, and anti‐vimentin rabbit monoclonal antibody were purchased from Cell Signaling Technology. Secondary biotin‐conjugated goat anti‐rabbit IgG antibody and goat anti‐mouse IgG antibody were obtained from Vector Laboratories.

### 2.5 Protein electrophoresis and immunoblotting

For one‐dimensional sodium dodecyl sulfate‐polyacrylamide gel electrophoresis (SDS‐PAGE), BALF samples were denatured in Laemmli buffer, boiled for 5 min and loaded onto 12% SDS‐PAGE gels. The separated proteins were transferred to polyvinylidene difluoride (PVDF) membranes (Millipore) at 100 V for an hour. After blocking the membrane with 5% skimmed milk in TBST, the membranes were reacted with primary anti‐human apoA‐I antibody (1:2000 dilution) at room temperature overnight. Biotin‐conjugated goat anti‐rabbit IgG was used as the secondary antibody (1:1000 dilution) for an hour at room temperature. Blots were visualized using 1:3000 streptoavidin‐Cy3 (Sigma‐Aldrich). Imaging and data analysis were performed with the ChemiDoc MP imaging system (Bio‐Rad).

### 2.6 Two‐dimensional gel electrophoresis (2‐DE)

2‐DE was performed as previously described.^8^, ^13^ Briefly, BALF samples were concentrated by acetone precipitation and resuspended in 8‐M urea, 4% 3‐[(3‐cholamidopropyl)‐dimethylammonio]‐1‐propanesulfonate (CHAPS), 65‐mM dithioerythritol, 0.1‐M acetic acid, pharmalyte (pH 3–10 for isoelectrofocusing; GE Healthcare), and a trace of bromophenol blue. Immobilized pH gradient (IPG) strips (pH 4–7, 18 cm; GE Healthcare) were rehydrated, and the concentrated BALF samples (100 µg of protein) were loaded onto the IPG strips. Isoelectrofocusing was performed in the IPG‐IEF Cool‐PhoreStar system (Anatech) at 20°C and terminated at 47 kV. The IPG strips were equilibrated in urea/SDS/Tris buffer for 30 min. The second‐dimensional run was performed on 10% polyacrylamide linear gels, with a constant current of 20–30 mA per gel at 20°C. The gels were stained with SYPRO Ruby Protein Gel Stain (Lonza Rockland).

### 2.7 Evaluation and identification of protein

We analyzed the BALF proteome in patients with acute and chronic HP in five cases of each with 2‐DE. The proteins were evaluated as described previously.^8^, ^13^ The gels were scanned using a FluoroPhoreStar 3000 image analysis system (Anatech) and analyzed with Progenesis PG220 Software (Nonlinear Dynamics Ltd.). The proteins were analyzed automatically using the spot detection feature of the software, with automatic warping and matching. Spot volumes were corrected for background and then normalized. The normalized spot volume was calculated as a percentage of the volume of each spot to the volumes of all spots in a gel. Proteins were identified by liquid chromatography nano‐electronspray ionization tandem mass spectrometry (LC‐nESI‐MS/MS) in the Laboratory of Cytometry and Proteome Research at Tokyo Medical and Dental University. LC separation was performed by the nano‐UHPLC system (BrukerDaltonics). Mass analysis was performed on a maXis‐4G‐CPR (BrukerDaltonics) mass spectrometer equipped with a nano‐ESI source.

### 2.8 Enzyme‐linked immunosorbent assay (ELISA)

ApoA‐I concentrations in BALF and serum were measured in a larger group of HP using an ELISA Kit (Abnova) according to the manufacturer's instructions. Chymase concentrations in BALF were also measured with an ELISA Kit (Cloud‐Clone Corp.) according to the manufacturer's instructions.

### 2.9 Analysis of the effects of enzyme inhibitor on apoA‐I degradation

Lipid‐free apoA‐I was isolated from human serum and purified as previously described.^19^ Lipid‐free apoA‐I was incubated with BALF from patients with acute and chronic HP (total protein, 3 µg). The mixtures were incubated at 37°C after adding chymostatin (chymase inhibitor, 80 µM; Sigma‐Aldrich), leupeptin (tryptase inhibitor, 100 µM; Sigma‐Aldrich) or 100‐µg/ml soybean trypsin inhibitor (Sigma‐Aldrich). The mixtures were separated by SDS‐PAGE and analyzed by immunoblotting with a primary anti‐human apoA‐I antibody.

### 2.10 Immunohistochemical analysis

The expression of apoA‐I in surgical lung biopsy specimens was evaluated. The specimens were stained with anti‐human apoA‐I antibody as described previously.^20^ Double immunohistochemical staining of MC_T_ (tryptase‐positive but chymase‐negative mast cells) and MC_TC_ (tryptase‐positive and chymase‐positive mast cells) were performed on both lung tissues and cytospin from BALF using the EnVision Double Stain Kit (Agilent Technologies), as described previously.^21^ Briefly, rehydration and antigen retrieval were performed in lung tissue sections and cultured cells were fixed with 4% paraformaldehyde. These were stained with both mouse anti‐human mast cell tryptase monoclonal antibody (1:12,000 dilution) and mouse anti‐human mast cell chymase (CC1) monoclonal antibody (1:2000 dilution). These samples were counterstained with Mayer's hematoxylin and mounted with a coverslip. For quantification of the positive staining cells and mast cells, cytospin slides were evaluated by counting 300 cells using the Image J software (National Institute of Health) and MCs in fibrotic areas of surgical lung biopsy specimens were counted in 20 random high power fields (×400) of fibrotic alveolar parenchyma.

### 2.11 Cell culture and TGF‐β1‐induced epithelial–mesenchymal transition (EMT)

A549 cells (Riken Cell Bank) were maintained in Dulbecco's modified Eagle's medium with 10% fetal bovine serum, 100‐U/ml penicillin and 100‐mg/ml streptomycin in a humidified incubator with 5% CO_2_ at 37°C. The cells (7.5 × 10^4^ per ml) were plated in six‐well culture plates and were synchronized overnight in serum‐free medium with 0.1% bovine serum albumin (BSA; Sigma‐Aldrich), chymostatin (80 µM), and either untreated lipid‐free apoA‐I (Merck) or chymase‐treated apoA‐I (final concentration, 50 µg/ml, each). The treatment of lipid‐free apoA‐I with recombinant mast cell chymase (Sigma‐Aldrich) was performed in 20‐mM Tris/HCl (pH 7.4) containing 150‐mM NaCl and 10‐mM CaCl_2_ at 37°C for 6 h. The full inhibition of chymase activity by chymostatin (100 µM) was confirmed with a Chymase Activity Assay Kit (Sigma‐Aldrich). Then, the cells were stimulated with TGF‐β1 (5 ng/ml; R&D Systems) for 48 h. Cellular morphological changes were evaluated using an inverted microscope (CKX53; Olympus).

### 2.12 Immunoblotting and quantitative real‐time polymerase chain reaction (PCR) of EMT markers

To evaluate the expressions of EMT markers (E‐cadherin, N‐cadherin, and vimentin), immunoblot analysis and quantitative PCR were performed. The cells were lysed in radioimmunoprecipitation assay buffer (Wako), and the cell lysates (10 µg) were separated by SDS/PAGE on 4%–15% gradient acrylamide gels and transferred to PVDF membranes. Anti‐E‐cadherin antibody (1:1000 dilution), anti‐N‐cadherin antibody (1:1000 dilution), anti‐vimentin antibody (1:1000 dilution), and anti‐β‐actin antibody (1:5000 dilution) were used as the primary antibodies.

The cells were lysed and total RNA was isolated using a Qiagen RNeasy Mini Kit (Qiagen) and the complementary DNA was synthesized from the total RNA using the SuperScript III First‐Strand Synthesis System for RT‐PCR (Invitrogen). Quantitative PCR was performed with SsoAdvanced Universal SYBR Green Supermix (Bio‐Rad Laboratories) using Bio‐Rad MiniOpticon machine (Bio‐Rad Laboratories). Primer sets were as follows: E‐cadherin forward: 5ʹ‐TGCACCAACCCTCATGAGTG‐3ʹ, E‐cadherin reverse: 5ʹ‐GTCAGTATCAGCCGCTTTCAG‐3ʹ; N‐cadherin forward: 5ʹ‐ATTGATGCTGACGATCCCAATGCC‐3ʹ, N‐cadherin reverse: 5ʹ‐TCAAGTCCAGCTGCCACTGTGATCA‐3ʹ; vimentin forward: 5ʹ‐AGAACCTGCAGGAGGCAGAAGAAT‐3ʹ, vimentin reverse: 5ʹ‐TTCCATTTCACGCATCTGGCGTT‐3ʹ; GAPDH forward: 5ʹ‐AGCAATGCCTCCTGCACCACCAAC‐3ʹ, GAPDH reverse: 5ʹ‐CCGCAGGGGGGCATCCACAGTCT‐3ʹ. Each messenger RNA (mRNA) expression was normalized by GAPDH.

### 2.13 Statistical analysis

All statistical analysis was performed with the GraphPad Prism software (version 7; GraphPad Software Inc.). Data were presented as medians with interquartile ranges or mean ± *SEM*. Comparisons between groups were analyzed with Mann–Whitney *U* tests or Fisher's exact test. Simple linear regression analyses were used to analyze correlations between variables and Spearman correlation coefficients (*r*) were calculated using these analyses. *p* < .05 was considered to be statistically significant.

## 3 RESULTS

### 3.1 Patient characteristics

Patient characteristics and clinical data are shown in Table 1. Thirty‐one patients with acute HP and 56 patients with chronic HP were enrolled. Five healthy volunteers (HV) were also enrolled as control. None of them had any past medical histories. All of the patients with acute and chronic HP underwent BAL, and 31 patients with chronic HP underwent surgical lung biopsy. Patients with chronic HP were significantly older and had a higher proportion of men than those with acute HP. Serum levels of C reactive protein and Krebs von den Lungen‐6 (KL‐6) were significantly lower in patients with chronic HP than those with acute HP. In BALF findings from patients with chronic HP, the cell density and the percentage of lymphocytes were significantly lower and the CD4/CD8 ratio was significantly higher than those in acute HP. There were no significant differences in the usage of statins or complications of dyslipidemia between acute and chronic HP. No patient was administered with any immunosuppressive treatment at the time of BAL.

**Table 1:** Patient characteristics

Characteristics	HV (*n* = 5)	Acute HP (*n* = 31)	Chronic HP (*n* = 56)	*p* value
Age (years)	32 (28–41)	55 (43–63)	61 (55–68)	.02*
Sex (male/female)	5/0	6/25	30/26	.003**
Smokers (no. of current/ex/never)	0/0/5	2/10/19	5/28/23	.08
Summer‐type HP/bird‐related HP	NA	5/26	5/51	.32
Usage of statins	0 (0%)	3 (9.6%)	1 (1.8%)	.13
Comorbidity				
Dyslipidemia	0 (0%)	3 (9.6%)	2 (3.6%)	.34
Cardiovascular disease	0 (0%)	2 (6.5%)	1 (1.8%)	.29
Laboratory findings				
Serum CRP (mg/dl)	NA	1.17 (0.17–2.0)	0.10 (0.09–0.30)	<.001***
Serum KL‐6 (U/ml)	NA	2505 (1308–5738)	1469 (1063–2892)	.03*
Serum SP‐D (ng/ml)	NA	322 (212–598)	232 (185–392)	.06
Pulmonary function test				
FVC (% predicted)	NA	80.2 (67.2–91.2)	81.5 (69.0–95.8)	.63
FEV_1.0_ (% predicted)	NA	83.0 (75.8–85.5)	82.9 (76.4–87.2)	.46
DLco (% predicted)	NA	50.0 (41.5–61.1)	60.8 (47.8–70.0)	.15
BALF profile				
Recovery rate (%)	71.3 (55.0–74.7)	57.0 (50.0–64.3)	56.7 (48.0–64.0)	.96
Cell density (×10^5^ per ml)	1.56 (1.14–2.05)	8.50 (4.60–11.2)	2.41 (0.92–4.28)	<.001***
Macrophages (%)	89.3 (73.6–93.9)	13.9 (9.1–24.0)	67.6 (38.5–82.5)	<.001***
Lymphocytes (%)	10.7 (5.4–16.4)	80.0 (61.0–87.3)	19.0 (10.1–50.0)	<.001***
Neutrophils (%)	0.0 (0.0–0.65)	1.45 (0.82–3.42)	1.75 (0.60–6.65)	.55
Eosinophils (%)	0.0 (0.0–0.0)	1.00 (0–3.82)	0.95 (0–1.7)	.71
CD4/CD8 ratio	NA	1.4 (0.3–2.67)	2.58 (1.01–5.01)	.005**

*Note*: Values are presented as the numbers or medians (25th and 75th percentiles). *p* values were derived from the analysis between acute HP and chronic HP.

Abbreviations: CRP, C reactive protein; DLco, diffusing capacity for carbon monoxide; FEV1.0, forced expiratory volume in 1 s; HP, hypersensitivity pneumonitis; HV, healthy volunteers; KL‐6, Kreb von den Lungen‐6; NA, not available; SP‐D, surfactant protein D; VC, vital capacity.

* *p* < .05.

** *p* < .01.

*** *p* < .001.

### 3.2 Striking difference of apoA‐I expression between acute and chronic HP

**Figure 1:**
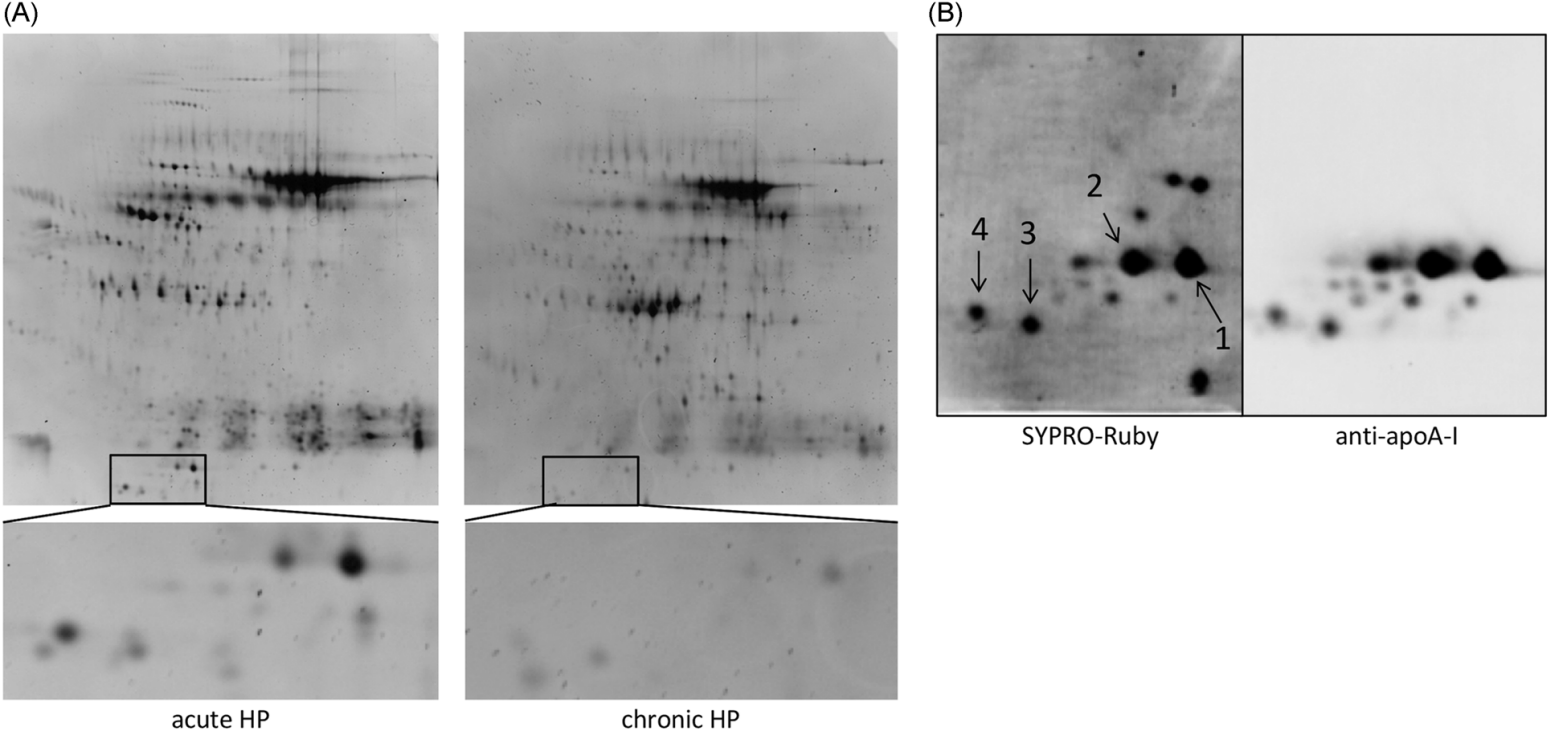
(A) Images of two‐dimensional electrophoresis separation of pooled BALF proteins from patients with acute and chronic HP. Magnifying apoA‐I lesion, acute HP has spots with higher intensities. (B) Focusing on apoA‐I lesion, proteins are visualized with SYPRO‐Ruby and apoA‐I antibody by immunoblotting. The proteins indicated by the numbers were also identified as apoA‐I by using LC‐nESI‐MS/MS. Spot numbers were referred to the annotations in Table S1. apoA‐I, apolipoprotein A‐I; BALF, bronchoalveolar lavage fluid; HP, hypersensitivity pneumonitis

2‐DE patterns of pooled BALF samples from five patients with acute and chronic HP are shown in Figure 1A,B. ApoA‐I was identified as the most differently expressed protein spot between acute and chronic HP by using LC‐nESI‐MS/MS (Table S1), showing that the expression of apoA‐I was lower in BALF from patients with chronic HP compared with acute HP. The expression of apoA‐I was confirmed with immunoblotting, in which 12 spots were detected and represented truncated apoA‐I (Figure 1B).

### 3.3 Difference of apoA‐I expression in clinical and histological patterns

**Figure 2:**
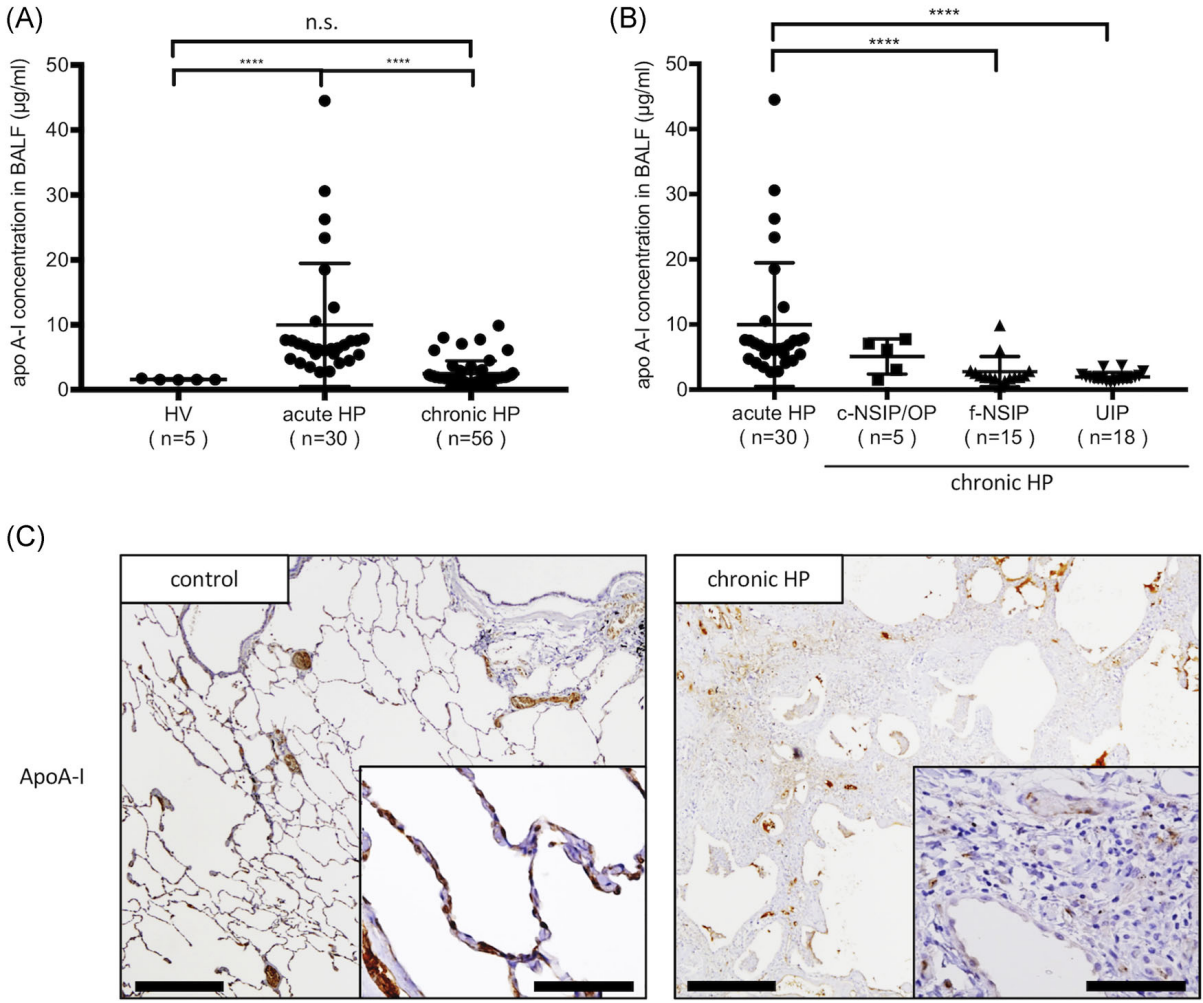
ELISA for measurement of apoA‐I concentrations in BALF and representative immunohistochemistry of apoA‐I proteins. (A) ApoA‐I concentrations in BALF from patients with HV, acute HP, and chronic HP. (B) ApoA‐I concentrations in BALF according to histological patterns of chronic HP. (C) Immunohistochemical staining of apoA‐I in lung tissues of control and chronic HP (UIP pattern) are shown. The expression of apoA‐I is lower in fibrotic lesions of chronic HP compared with normal lung tissues. Bars showed the mean values ± *SEM*. apoA‐I, apolipoprotein A‐I; BALF, bronchoalveolar lavage fluid; ELISA, enzyme‐linked immunosorbent assay; HP, hypersensitivity pneumonitis; HV, healthy volunteers; c‐NSIP, cellular nonspecific interstitial pneumonia; f‐NSIP, fibrotic nonspecific interstitial pneumonia; OP, organizing pneumonia; UIP, usual interstitial pneumonia; n.s., not significant. Scale bars = 500 µm and 50 µm in insets. ****p < .0001

To confirm the results of the BALF proteome, apoA‐I concentrations in BALF were measured in a larger group of HP using ELISA (Figure 2). The concentrations of apoA‐I were significantly lower in BALF from patients with chronic HP compared with acute HP (*p* < .0001, 2.50 ± 0.27 and 9.99 ± 1.73 µg/ml, respectively; Figure 2A). No significant difference between the concentrations of apoA‐I in BALF from chronic HP and those with HV was observed (*p* = .18). The concentrations of apoA‐I in BALF were significantly different according to histological subtypes, showing the lower levels in chronic HP with f‐NSIP pattern or UIP pattern compared to acute HP (Figure 2B). There was no significant correlation between the concentrations of apoA‐I in serum and BALF of acute and chronic HP (*r* = .01; *p* = .92; Figure S1).

Immunohistochemical localization of apoA‐I in normal lung parts in the resected specimens of lung cancer as control and lung tissues from chronic HP are shown in Figures 2 and S2. In control tissues, apoA‐I was expressed in type I and type II AECs and alveolar macrophages (Figure 2C). The expression of apoA‐I was decreased in the fibrotic lesions of patients with chronic HP compared with control tissues.

### 3.4 Higher level of chymase in BALF of HP patients and its degradative effect on apoA‐I

**Figure 3:**
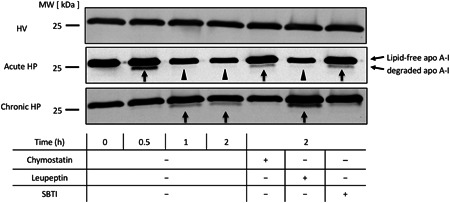
Immunoblotting for apoA‐I in the mixture of lipid‐free apoA‐I and pooled BALF from HV and patients with acute and chronic HP. Fragmentation (arrow) and degradation (arrowhead) of apoA‐I were observed over a time‐course. The degradation of apoA‐I was inhibited by chymostatin and SBTI, but not by leupeptin, in acute and chronic HP. apoA‐I, apolipoprotein A‐I; BALF, bronchoalveolar lavage fluid; HP, hypersensitivity pneumonitis; HV, healthy volunteers; SBTI, soybean trypsin inhibitor

**Figure 4:**
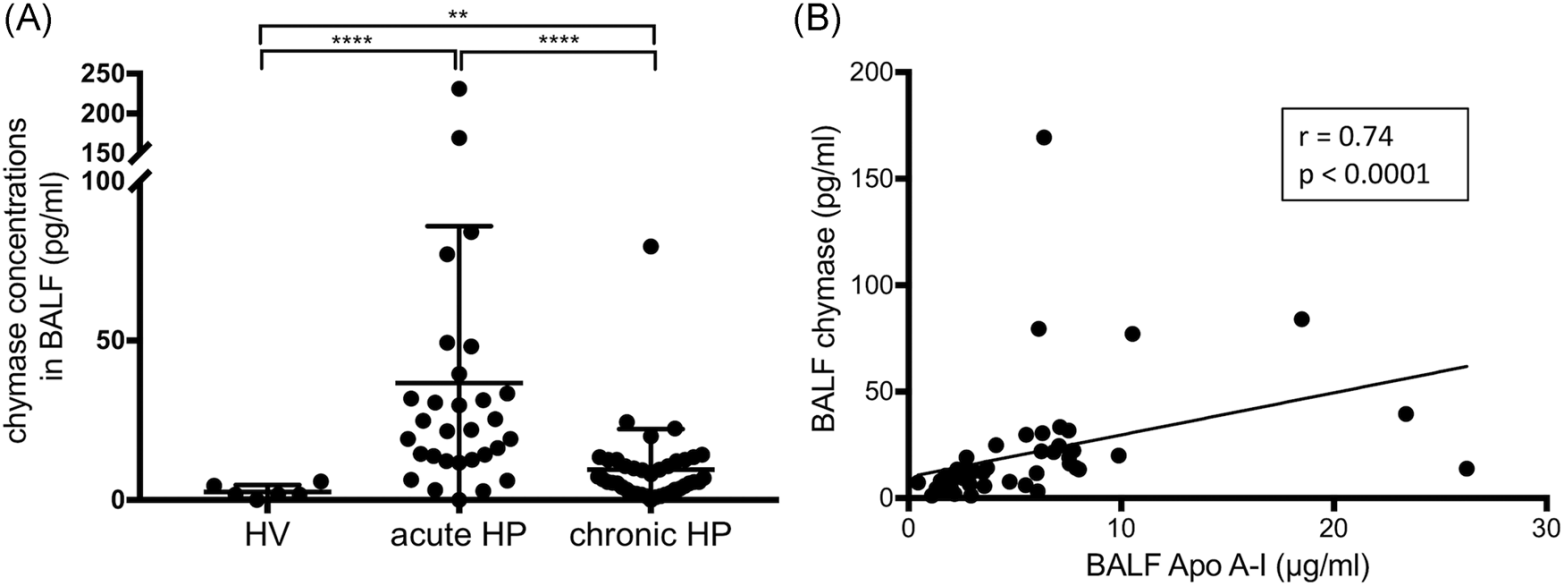
(A) The chymase concentration in BALF of HV, acute HP, and chronic HP. Acute HP, 36.7 ± 8.98 pg/ml; chronic HP, 9.51 ± 2.02 pg/ml; and HV, 2.59 ± 0.87 pg/ml. (B) Relationship between the concentration of chymase and the concentration of apoA‐I in BALF from patients with acute and chronic HP. apoA‐I, apolipoprotein A‐I; BALF, bronchoalveolar lavage fluid; HP, hypersensitivity pneumonitis; HV, healthy volunteers. Bars showed the mean ± *SEM*. **p < .01, ****p < .0001

To examine whether apoA‐I is degraded by BALF of HP patients, lipid‐free apoA‐I was incubated with BALF samples. Immunoblot analysis using anti‐human apoA‐I antibody demonstrated that lipid‐free apoA‐I was fragmented and degraded after the mixture with BALF from patients with acute and chronic HP (Figure 3). In contrast, there was no fragmentation and degradation using BALF from HV. Interestingly, the degradation of lipid‐free apoA‐I incubated with BALF from acute and chronic HP for 2 h was inhibited by chymostatin (chymase inhibitor) and soybean trypsin inhibitor (trypsin and chymotrypsin inhibitor). However, leupeptin (tryptase inhibitor) did not inhibit degradation. To confirm the immunoblotting results, chymase concentrations in BALF were measured using ELISA. The chymase concentrations in BALF were significantly higher in acute and chronic HP compared with HV (36.70 ± 8.98, 9.51 ± 2.02, and 2.59 ± 0.87 pg/ml, respectively; Figure 4A). Furthermore, a positive correlation between the concentration of chymase and the concentration of apoA‐I was observed in BALF from patients with acute and chronic HP (*r* = .74; *p* < .0001; Figure 4B).

### 3.5 Increased appearance of MC_TC_ in BALF and lung tissues of chronic HP

**Figure 5:**
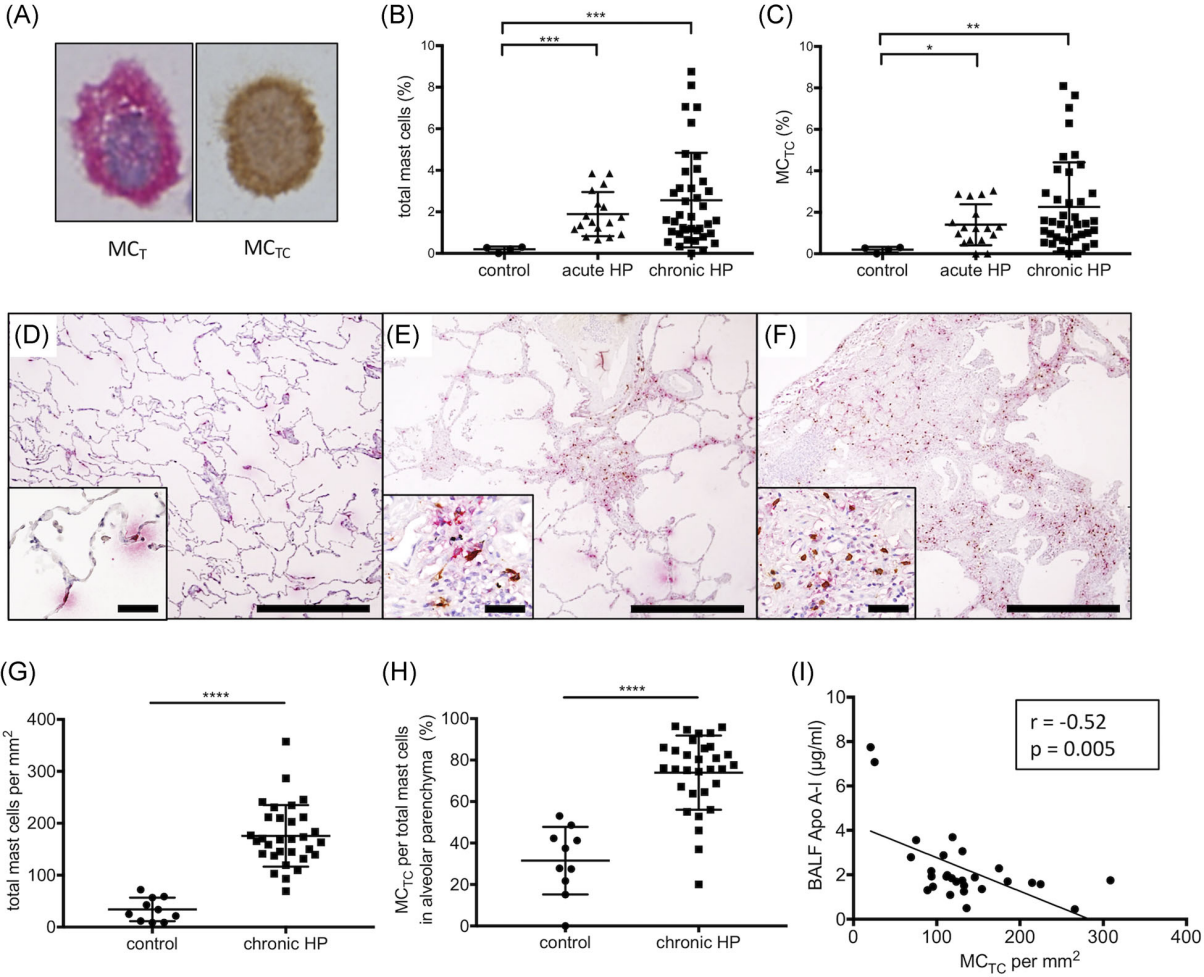
Tryptase‐positive mast cells (MC_T_) and chymase‐positive mast cells (MC_TC_) in the BALF and lungs. (A) Representative images of immunohistochemical double staining for MC_T_ (permanent red) and MC_TC_ (DAB, brown). (B) The percentage of total mast cells and (C) MC_TC_ in BALF from patients with acute HP, chronic HP, and HV. (D–F) Representative images of MC_T_ and MC_TC_ in alveolar parenchyma from normal lung tissues, acute HP, and chronic HP (UIP‐like lesion) are shown. (G) Total mast cell density per mm^2^ and (H) the proportion of MC_TC_ in alveolar parenchyma from normal lung and fibrotic alveolar parenchyma from chronic HP. (I) Relationship between MC_TC_ density in alveolar parenchyma and apoA‐I concentrations in BALF in chronic HP (Spearman's correlation coefficient *r* = −.52; *p* = .005). apoA‐I, apolipoprotein A‐I; BALF, bronchoalveolar lavage fluid; DAB, 3,3′‐diaminobenzidine; HP, hypersensitivity pneumonitis; HV, healthy volunteers; UIP, usual interstitial pneumonia. Bars showed the mean ± *SEM*. Scale bars = 500 µm and 50 µm in insets. *p < .05, **p < .01, ***p < .001, ****p < .0001

Next, we evaluated the densities and phenotypes of mast cell populations in BALF and lung tissues from patients with chronic HP and normal controls, because chymase is mainly released by the activation/degranulation of mast cells in fibrotic lung disease.^22^ Immunohistochemical images of mast cells, including tryptase+/chymase− mast cells (MC_T_) and tryptase+/chymase+ mast cells (MC_TC_), are shown in Figure 5. The percentage of total mast cells in BALF was significantly higher in acute and chronic HP compared with HV (*p* < .01; 1.89 ± 0.25%, 2.56 ± 0.36%, and 0.20 ± 0.07%, respectively; Figure 5B). In addition, the percentage of MC_TC_ in BALF was significantly higher in acute and chronic HP compared with HV (*p* = .01; 1.40 ± 0.24%, 2.26 ± 0.34%, 0.19 ± 0.06%, respectively; Figure 5C). Immunohistochemistry shows that more MC_TC_ were seen in inflammatory and fibrotic alveolar parenchyma of acute and chronic HP compared with normal alveolar parenchyma (Figure 5D). Because only two cases underwent surgical lung biopsy in acute HP, mast cells in alveolar parenchyma of acute HP were not evaluated. The number of total mast cells was significantly higher in fibrotic alveolar parenchyma of chronic HP compared with normal lung tissues (*p* < .0001; 175 ± 10.7 and 34.0 ± 7.2 cells/mm^2^, respectively; Figure 5E) and the ratio of MC_TC_/total mast cells was significantly higher (*p* < .0001; 74.0 ± 3.21% and 31.4 ± 5.15%; Figure 5F). Furthermore, an inverse correlation between the number of lung MC_TC_ and the concentration of apoA‐I in BALF was observed in patients with chronic HP (*r* = −.52; *p* = .005; Figure 5G). These findings suggested that more MC_TC_ may contribute to the degradation of apoA‐I in the fibrotic lesions.

### 3.6 Mast cell chymase treatment inhibits TGF‐β1‐induced EMT in A549 cells through the degradation of apoA‐I

**Figure 6:**
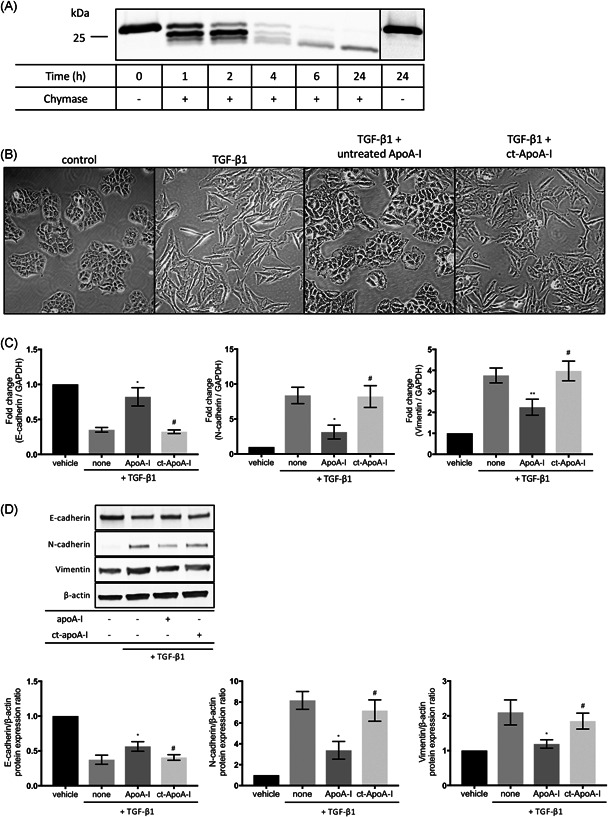
Inhibition of TGF‐β1‐induced EMT in A549 cells through the degradation of apoA‐I. (A) Immunoblotting of apoA‐I (1 mg/ml) in the absence or presence of chymase (0.001 U/ml BTEE) for the indicated times. (B) Untreated apoA‐I inhibits TGF‐β1‐induced morphological changes, but not chymase‐treated apoA‐I. The expression changes of (C) mRNA and (D) protein in EMT‐related markers, such as E‐cadherin, N‐cadherin, and vimentin, following TGF‐β1‐induced EMT in untreated apoA‐I or chymase‐treated apoA‐I. apoA‐I, apolipoprotein A‐I; ct‐ApoA‐I, chymase‐treated apoA‐I; BTEE, *N*‐benzoyl‐l‐tyrosine ethyl ester; EMT, epithelial–mesenchymal transition; mRNA, messenger RNA; TGF‐β1, transforming growth factor β. Six independent experiments were performed. Data are represented as the mean ± *SEM*. *p < .05, **p < .01 (untreated apoA‐I vs. TGF‐β1), ^#^ *p* < .05 (chymase‐treated apoA‐I vs. untreated apoA‐I)

To evaluate the impact of degradation of apoA‐I on lung fibrogenesis, we investigated whether chymase‐treated apoA‐I could have an effect on antifibrotic properties in TGF‐β1‐induced EMT. Treatment of lipid‐free apoA‐I with recombinant human mast cell chymase resulted in the degradation of apoA‐I in a time‐dependent manner (Figure 6A). After stimulation with TGF‐β1 in condition with untreated apoA‐I or chymase‐treated apoA‐I, the morphological characteristics and expression of EMT markers, including E‐cadherin, N‐cadherin, and vimentin, were evaluated. A549 cells were converted from epithelial phenotype to mesenchymal phenotype (spindle shape) in response to TGF‐β1. The shape of the epithelial phenotype was restored in the untreated apoA‐I condition, but not in response to the chymase‐treated apoA‐I (Figure 6B). Immunoblotting and quantitative PCR analysis also showed that coincubation with the untreated apoA‐I and TGF‐β1 significantly suppressed TGF‐β1‐induced downregulation of the epithelial marker (E‐cadherin) and upregulation of the mesenchymal markers (N‐cadherin and vimentin) in mRNA and protein expression levels, but this trend was not observed in the coincubation with chymase‐treated apoA‐I and TGF‐β1 (Figure 6C,D). Taken together, these data suggest that the degradation of lipid‐free apoA‐I with chymase may attenuate the protective effect of apoA‐I in EMT.

## 4 DISCUSSION

In this study, we identified that apoA‐I concentrations in BALF were higher in acute HP compared with chronic HP and controls. Similarly, chymase concentrations in BALF were higher in acute and chronic HP compared with controls. The number of MC_TC_, which is a main source for chymase in the lung, was increased in BALF of acute and chronic HP compared with controls. In vitro studies using A549 cells demonstrated that apoA‐I inhibited the TGF‐β1‐induced EMT. Considering apoA‐I has anti‐inflammatory and antifibrotic effect as previous papers,^12^, ^14^ our results suggest that apoA‐I might be increased against lung inflammation in HP and the degradation of apoA‐I by mast cell chymase could be important for the pathogenesis of chronic HP.

The differences in the protein profile of BALF between acute and chronic HP, potentially reflecting the pathogenesis of pulmonary fibrosis in HP, have not been fully investigated.^9^ We first identified apoA‐I as the most differently expressed protein between acute and chronic HP using 2‐DE and LC‐nESI‐MS/MS. ApoA‐I is synthesized predominantly in the liver and small intestine, but scarcely in the lung.^10^, ^23^ In our studies, apoA‐I protein was detected in both human BALF and AECs. We previously revealed that BALF apoA‐I was higher in Stage I sarcoidosis compared with that of Stage IV, suggesting that BALF apoA‐I might increase against lung inflammation in sarcoidosis.^13^ ApoA‐I has immunomodulatory properties against inflammation.^12^ ApoA‐I can modulate adaptive immunity by attenuating Th1/Th17 immune responses in methylated BSA‐induced arthritis mice model.^24^ ApoA‐I can attenuate antigen presentation and T‐cell activation by the decrease of major histocompatibility complex class II proteins in ovalbumin allergic mice model.^25^ Acute immune responses against inhalation of causative antigens is also important for the pathogenesis of HP, including antigen presentation by dendritic cells and Th1/Th17 responses.^2^, ^26^, ^27^ Taking together, the increase of apoA‐I concentration in BALF may reflect an anti‐inflammatory response locally against inhalation of causative antigens in HP, although the direct secretion of apoA‐I by lung cells into BALF has not yet been demonstrated.^12^ In contrast, the expression of apoA‐I was significantly lower in BALF from patients with chronic HP compared with acute HP. In addition, the expression of apoA‐I was lower in the fibrotic alveolar parenchyma of chronic HP compared with normal lung tissues (Figure 3). These results are consistent with the previous paper which showed that apoA‐I was decreased in BALF from patients with IPF.^14^

Mast cells contribute to innate immunity, adaptive immunity, and tissue repair and produce various mediators involved in inflammatory responses.^28^, ^29^, ^30^ Mast cells also play a role in the pathogenesis of lung fibrosis.^22^, ^29^, ^30^, ^31^, ^32^, ^33^, ^34^, ^35^ Among mast cell mediators, chymase is one of the mediators associated with fibrosis. Mast cells have heterogeneity and are classified into MC_T_ and MC_TC_.^28^, ^36^ Andersson et al.^21^ reported that IPF lungs had alteration of mast cell populations and more MC_TC_. In vivo experiment with a hamster model of bleomycin‐induced fibrosis revealed that chymase inhibitor reduced pulmonary fibrosis.^22^ Previous experimental fibrosis model also demonstrated that mature mast cells accumulated and MC_TC_ was increased in fibrotic lesions in the chronic phase of pulmonary fibrosis.^35^ A significant increase of mast cells was found in BALF and lung tissues from HP as well^34^, ^37^ and degranulation of mast cells plays an important role in the fibrotic process of HP.^38^, ^39^, ^40^ In the present study, total mast cells and MC_TC_ were increased in chronic HP lungs and chymase concentrations were higher in BALF from chronic HP patients compared with controls, suggesting that mast cell chymase may be involved in the pathogenesis of chronic HP.

In BALF of HP patients, chymase concentrations were positively correlated with apoA‐I concentrations. Although chymase is inactivated in mast cell granules, the enzymatic effects of chymase are activated in the loci of the tissue microenvironment after mast cell degranulation.^30^ Chymase is known to be a pro‐inflammatory mediator followed by activation of various inflammatory cytokines.^30^ Considering that the increase of apoA‐I concentration in BALF might reflect lung inflammation against the inhalation of causative antigens, BALF chymase might be increased by lung inflammation in HP.

In contrast, the concentrations of apoA‐I were lower in BALF from patients with chronic HP compared with acute HP. Although Kim et al.^14^ reported that apoA‐I concentrations is lower in BALF from patients with IPF, the reason remains poorly understood. Previous studies reveal that mast cell chymase, which is a chymotrypsin serine protease, plays an important role in the dynamics of apoA‐I.^19^, ^41^, ^42^ The degradation of apoA‐I incubated with BALF from HP patients was inhibited by chymostatin (chymase inhibitor) and soybean trypsin inhibitor, which have chymotryptic inhibitory activity. Furthermore, an inverse correlation between the number of lung MC_TC_ and apoA‐I concentrations in BALF was observed in chronic HP (Figure 5G), although no significant correlation between lung MC_TC_ and BALF chymase concentrations was observed in chronic HP (data not shown, *r* = .02; *p* = .92). Because the proteolytic ability of released chymase is rapidly inactivated by antipeptidase, including endogenous protease inhibitors of serpin type,^30^ the impact of chymase on the degradation of apoA‐I might be more active in the loci of pulmonary fibrosis. Taken together, our results suggest that chymase produced by mast cells may play an important role in the degradation of apoA‐I in chronic HP.

Lipid‐free apoA‐I inhibited EMT in this study. In contrast, chymase‐treated apoA‐I lost the ability to inhibit EMT in A549 cells (Figure 6). Baek et al.^43^ reported that apoA‐I also had antifibrotic function with the inhibition of EMT in A549 cells, which is consistent with our results. Previous study revealed that the degradation of apoA‐I led to lose its protective ability. In vitro experiments with human coronary artery endothelial cell lines and human macrophage foam cells demonstrated that the degradation of lipid‐free apoA‐I by mast cell chymase resulted in the loss of anti‐inflammatory function, as evidenced by the increases of inflammatory cytokines after lipopolysaccharide stimulation.^22^ However, our previous study showed that EMT was a part of the fibrotic process of chronic HP.^44^ These results indicate that the degradation of apoA‐I in BALF may influence EMT in the pathogenesis of chronic HP.

Our study has several limitations. First, this study includes a relatively small number of sample. Second, it has not been clarified whether the administration of apoA‐I attenuated pulmonary fibrosis in experimental animal models of chronic HP. Several preclinical animal studies revealed that the administration of apoA‐I or apoA‐I mimetic peptides attenuate disease severity in various lung disease models.^12^, ^45^ Although inhalational administration of apoA‐I or apoA‐I mimetic peptides could be a therapeutic option for HP patients, further studies are needed to evaluate these therapeutic approaches.

In conclusion, this study demonstrated that apoA‐I was reduced in BALF from patients with chronic HP and mast cell chymase attenuated the protective effect of apoA‐I in terms of fibrosis. The loss of balance between apoA‐I and mast cell chymase might be also associated with the pathogenesis of chronic HP. Thus, apoA‐I could be a crucial molecule associated with lung fibrogenesis of HP.

## CONFLICT OF INTERESTS

The authors declare that there are no conflict of interests.

## AUTHOR CONTRIBUTIONS

Yasunari Miyazaki had full access to all of the data in the study and takes responsibility for the integrity of the data and the accuracy of the data analysis. Yasunari Miyazaki and Yukihisa Inoue contributed to the conception and design of the study;  Yukihisa Inoue, Tsukasa Okamoto, Takayuki Honda, and Yasunari Miyazaki contributed to the drafting of the submitted article; Tsukasa Okamoto, Takayuki Honda, and Yasunari Miyazaki contributed to the critical revision of the manuscript for important intellectual property; all authors contributed to the analysis and interpretation of the data.

## Supporting information

Supporting information.

Supporting information.

Supporting information.
