# Supplementary material for: Disruption in the balance between apolipoprotein A‐I and mast cell chymase in chronic hypersensitivity pneumonitis
Source: Immun Inflamm Dis. 2020 Oct 4;8(4):659–71. doi: 10.1002/iid3.355 (PMC7654418; doi:10.1002/iid3.355)
Supplement: Supplementary file 1 — Supporting information. [file IID3-8-659-s001.docx]

**Additional File**

**Disruption in the balance between apolipoprotein A-I and mast cell chymase in chronic hypersensitivity pneumonitis**

Yukihisa Inoue^1)^, Tsukasa Okamoto^1)^, Takayuki Honda^1)^, Yoshihisa Nukui^1)^, Takumi Akashi^2)^, Tamiko Takemura^3)^, Minoru Tozuka^4)^, Yasunari Miyazaki^1)^

AFFILIATIONS:

1) Department of Respiratory Medicine, Tokyo Medical and Dental University

2) Department of Pathology, Tokyo Medical and Dental University

3) Department of Pathology, Japan Red Cross Centre

4) Department of Analytical Laboratory Chemistry, Graduate School of Medical and Dental Sciences, Tokyo Medical and Dental University

CORESPONDENCE:

Yasunari Miyazaki, M.D. Ph.D.

Department of Respiratory Medicine, Tokyo Medical and Dental University

1-5-45, Yushima, Bunkyo-ku, Tokyo 113-8510, Japan

Telephone number: +81-3-5803-5954

E-mail: [miyazaki.pilm@tmd.ac.jp](mailto:miyazaki.pilm@tmd.ac.jp)

**Additional file – Results**

**Table S1**. Identification of the proteins indicated by the numbers (Fig. 1). Five samples per group (acute and chronic HP) were analysed. Spot no.: refer to the annotations in Fig. 1B.

Values are given as the numbers or medians (interquartile ranges). AC, accession number from the SWISS-PROT database; HP, hypersensitivity pneumonitis.

| Spot no. | AC | Protein | Theoretical MW (kDa) / pI | Normalization volume of acute HP, ×10^-3^ | Normalization volume of chronic HP, ×10^-3^ | p value |
| --- | --- | --- | --- | --- | --- | --- |
| 1 | P-02647 | Apolipoprotein A-I | 28 / 5.27 | 457 (302-718) | 107 (25-218) | 0.02 |
| 2 |  |  | 28 / 5.17 | 349 (207-470) | 67 (25-228) | 0.008 |
| 3 |  |  | 24 / 5.04 | 137 (44-319) | 26.5 (5-83) | 0.03 |
| 4 |  |  | 24 / 5.04 | 161 (30-270) | 25 (2-37) | 0.01 |

**Figure S1.** Relationship of apolipoprotein A-I (apoA-I) concentrations between BALF and serum. BALF, bronchoalveolar lavage fluid.

**Figure S2.** Representative immunohistochemistry of apoA-I in normal alveolar parenchyma from patients with chronic HP with UIP patterns. Haematoxylin and eosin staining (a), apoA-I staining (b) are shown. Scale bars: 150 µm and 15 µm in inserts. ApoA-I, apolipoprotein A-I; HP, hypersensitivity pneumonitis; UIP, usual interstitial pneumonia.
