# Supplementary figures and images for: Disruption in the balance between apolipoprotein A‐I and mast cell chymase in chronic hypersensitivity pneumonitis
Source: Immun Inflamm Dis. 2020 Oct 4;8(4):659–71. doi: 10.1002/iid3.355 (PMC7654418; doi:10.1002/iid3.355)

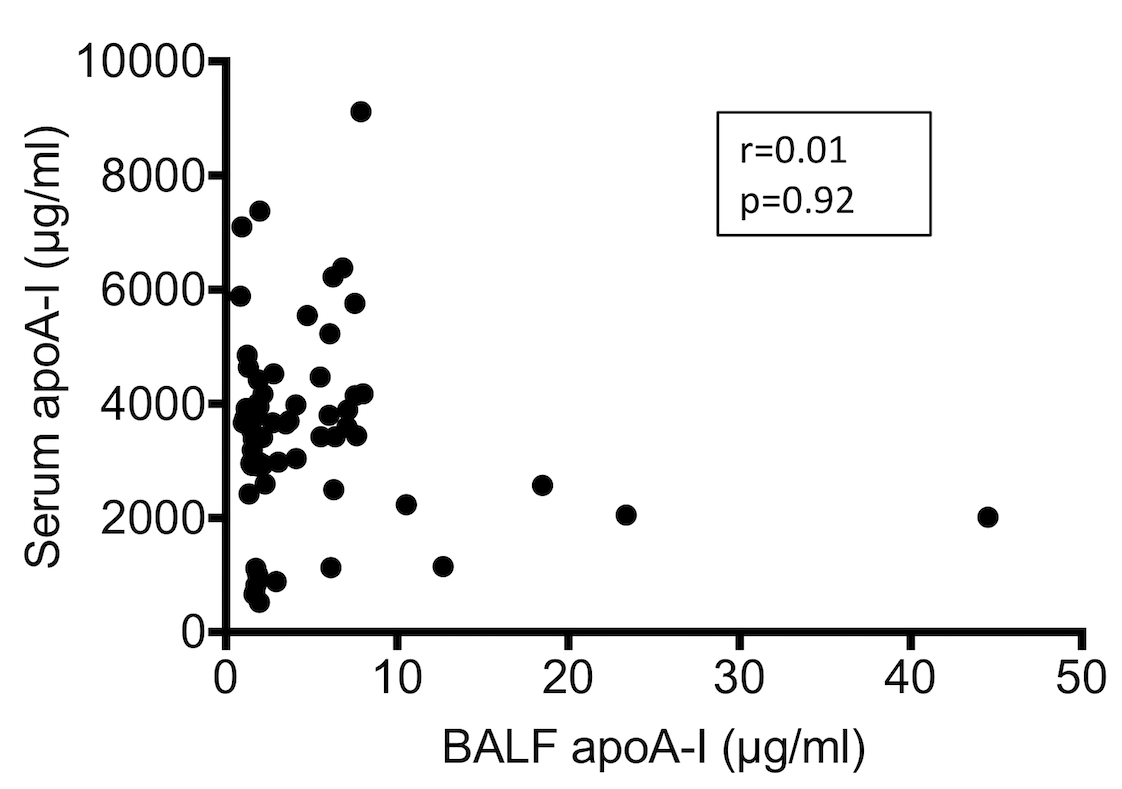

Supplement: Supplementary file 2 — Supporting information. [file IID3-8-659-s002.tiff]

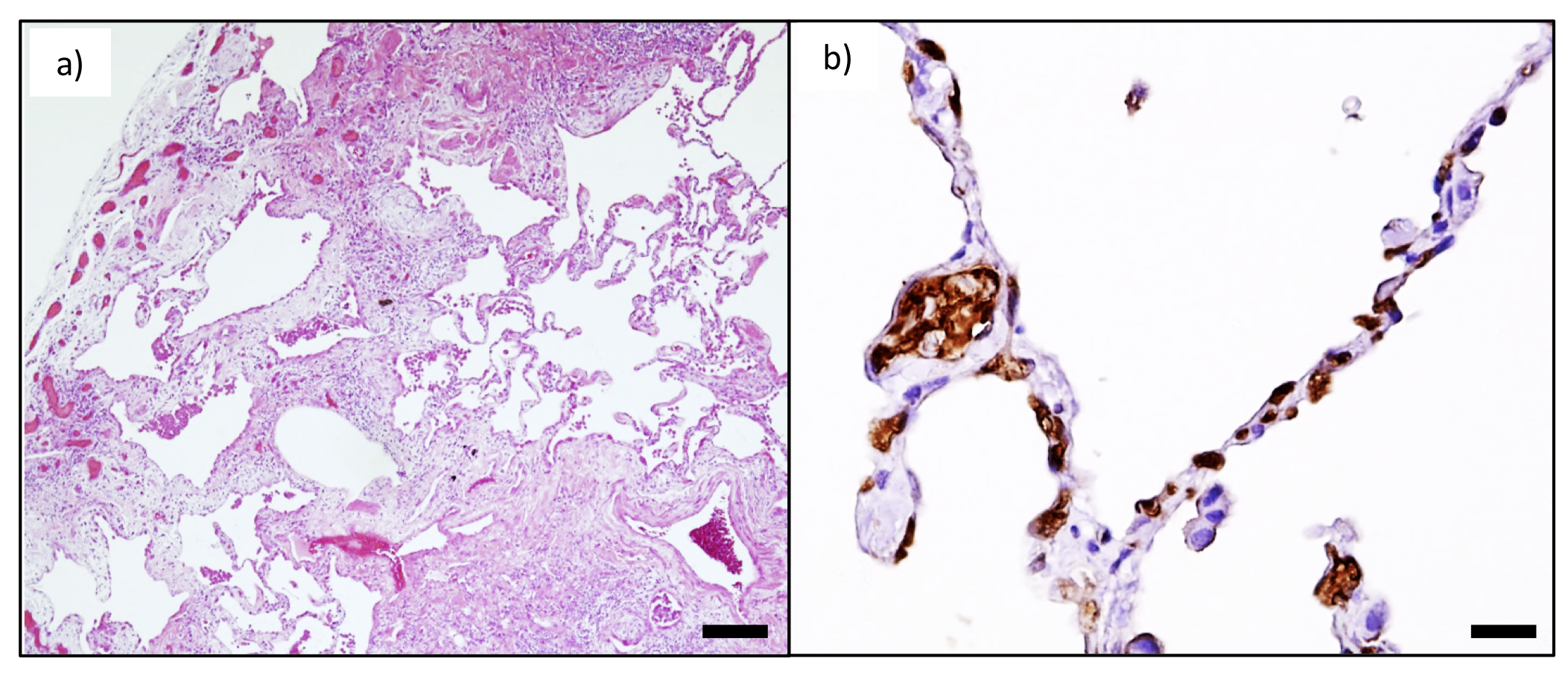

Supplement: Supplementary file 3 — Supporting information. [file IID3-8-659-s003.tiff]
